# Hexa-μ-acetato-1:2κ^4^ *O*,*O*′;1:2κ^2^ *O*:*O*;2:3κ^4^ *O*,*O*′;2:3κ^2^ *O*:*O*-bis­(4,4′-dimethyl-2,2′-bi­pyridine)-1κ^2^ *N*,*N*′;3κ^2^ *N*,*N*′-2-calcium-1,3-dizinc

**DOI:** 10.1107/S1600536813030122

**Published:** 2013-11-09

**Authors:** Avijit Pramanik, Frank R. Fronczek, Ramaiyer Venkatraman, Md. Alamgir Hossain

**Affiliations:** a Department of Chemistry and Biochemistry, Jackson State University, Jackson, MS 39217, USA; b Department of Chemistry, Louisiana State University, Baton Rouge, LA 70803, USA

## Abstract

In the centrosymmetric trinuclear Zn^II^⋯Ca^II^⋯Zn^II^ title complex, [CaZn_2_(CH_3_COO)_6_(C_12_H_12_N_2_)_2_], the Ca^II^ ion lies on an inversion centre and is octa­hedrally coordinated by six acetate O atoms. The Zn^II^ ion is coordinated by two N atoms from a bidentate di­methyl­bipyridine ligand and three O atoms from acetate ligands bridging to the Ca^II^ ion, leading to a distorted square-pyramidal coordination sphere. The Zn⋯Ca distance is 3.4668 (5) Å.

## Related literature

For a review of the coordination chemistry of metal carboxyl­ates, see: Rao *et al.* (2004 ▶). For applications of metal complexes in anion binding, see: Saeed *et al.* (2010 ▶); Mendy *et al.* (2010 ▶). For multimetallic complexes involving azide, cyanide, isocyanate, isothiocyanate, hydroxide, oxide and carboxylate anions, see: Herold & Lippard (1997 ▶). For coordin­ation modes of carboxyl­ate ions acting as bidentate ligands in metalloenzymes, see: Voegtli *et al.* (2000 ▶). For details of the synthesis, see: Hossain *et al.* (2010 ▶).
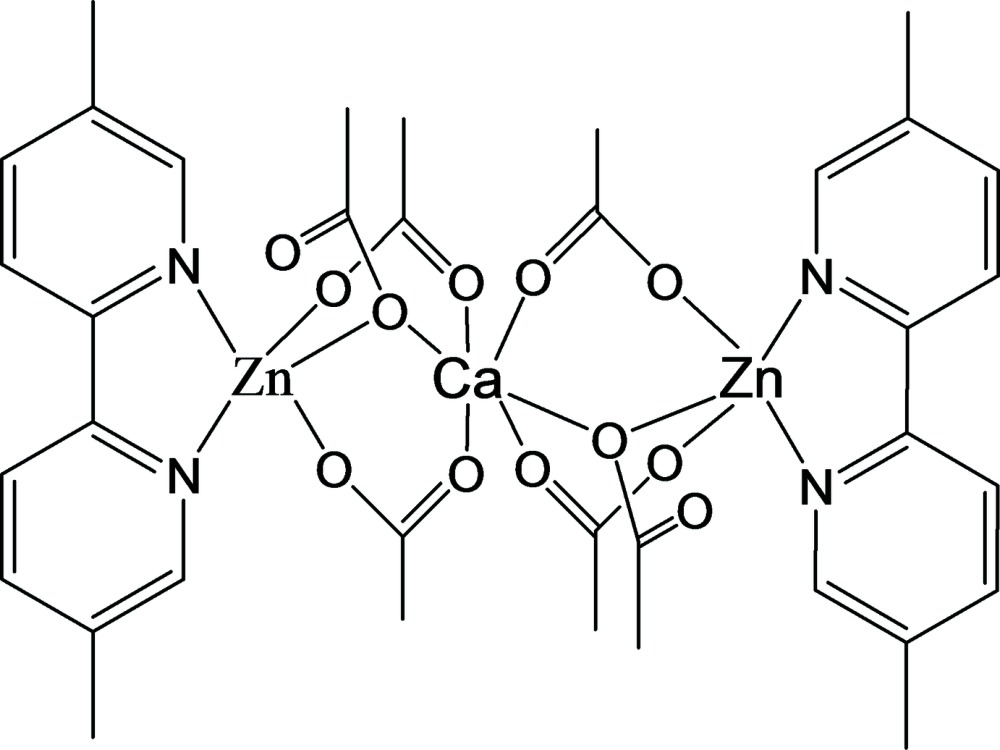


## Experimental

### 

#### Crystal data

[CaZn_2_(C_2_H_3_O_2_)_6_(C_12_H_12_N_2_)_2_]
*M* *_r_* = 893.56
Triclinic, 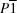

*a* = 8.3356 (10) Å
*b* = 8.841 (1) Å
*c* = 13.696 (2) Å
α = 74.103 (7)°
β = 83.263 (6)°
γ = 83.560 (7)°
*V* = 960.6 (2) Å^3^
*Z* = 1
Mo *K*α radiation
μ = 1.45 mm^−1^
*T* = 100 K
0.34 × 0.32 × 0.27 mm

#### Data collection

Nonius KappaCCD diffractometer
Absorption correction: multi-scan (*SCALEPACK*; Otwinowski & Minor, 1997 ▶) *T*_min_ = 0.639, *T*_max_ = 0.699
16096 measured reflections
8962 independent reflections
7497 reflections with *I* > 2σ(*I*)
*R*_int_ = 0.025

#### Refinement

*R*[*F* ^2^ > 2σ(*F* ^2^)] = 0.034
*wR*(*F* ^2^) = 0.086
*S* = 1.05
8962 reflections
256 parameters
H-atom parameters constrained
Δρ_max_ = 0.65 e Å^−3^
Δρ_min_ = −0.78 e Å^−3^

### 

Data collection: *COLLECT* (Nonius, 2000 ▶); cell refinement: *SCALEPACK* (Otwinowski & Minor, 1997 ▶); data reduction: *DENZO* (Otwinowski & Minor, 1997 ▶) and *SCALEPACK*; program(s) used to solve structure: *SIR97* (Altomare *et al.*, 1999 ▶); program(s) used to refine structure: *SHELXL97* (Sheldrick, 2008 ▶); molecular graphics: *ORTEP-3 for Windows* (Farrugia, 2012 ▶); software used to prepare material for publication: *SHELXL97*.

## Supplementary Material

Crystal structure: contains datablock(s) global, I. DOI: 10.1107/S1600536813030122/fj2647sup1.cif

Structure factors: contains datablock(s) I. DOI: 10.1107/S1600536813030122/fj2647Isup2.hkl

Additional supplementary materials:  crystallographic information; 3D view; checkCIF report

## Figures and Tables

**Fig. 1.**
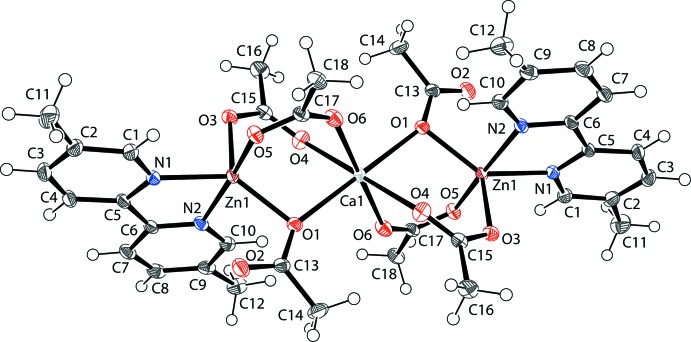
ORTEP drawings of the crystal structure and atomic numbering scheme of trinuclear ZnII—CaII—ZnII complex. Ellipsoids are set at the 50% probability level.

## supplementary crystallographic information

### 1. Comment

Multimetallic coordination complexes have many practical applications in a number of areas including molecular magnetism, crystal engineering, catalysis, gas storage, ion exchange, nonlinear optics, and biomimetic materials (Rao *et al.*, 2004). In general, multimetallic complexes invoves azido, cyano, isocyanate, isothiocyanate, hydroxo, oxo and carboxylate ions (Herold & Lippard, 1997). Multimetallic complexation is also important in many biological systems; for example, in metalloenzymes where carboxylate ions serve as bidentate ligands showing three different coordination modes: *syn-syn, syn-anti* and *anti-anti* (Voegtli *et al.*, 2000). However, as compared to traditional mononuclear metal complexes, the chemistry of multimetallic complexes with well established structures is much under developed. In our continuing interests in dinuclear metal complexes (Saeed *et al.*, 2010; Mendy, *et al.*, 2010) for anion binding, we attempted to obtain zinc complex of mixed ligands with 4,4'dimethyl-2,2'-bipyridine and hexa *N*-substituted (*p*-cyano benzamido) *para* xylyl based octa-aza cryptand. However, single-crystal structure analysis reveals the formation of the title compound. The source of calcium could be a contaminant from the reagents used for this reaction. Herein, we present a structural characterization of 4,4'-dimethyl-2,2'-bipyridine coordinated heterotrinuclear Zn^II^—Ca^II^—Zn^II^ hexa-carboxylate complex.

The heteronuclear complex is crystallized in triclinic P-1 space group, with one calcium ion, two zinc ions, six acetate and two 4,4'-dimethyl-2,2'-bipyridine groups. As can be seen in Figure 1, one calcium ion is located at the center surrounded by two zinc ions and six acetate ions. Two zinc ions from opposite sides are linearly coordinated with the central calcium ion forming trinuclear Zn^II^–Ca^II^–Zn^II^ backbone, while six acetate ions are coordinated with six Ca–O bonds. In addition, each zinc is hexacoordinated with two N atoms from one bipyridine and four O atoms from four six acetates, forming a centrosymmetric complex. In the complex, four acetates serve as bidentate ligands to coordinate with both calcium and zinc ions, while each of other two serves as a monodentate ligand for a single zinc ion. Both Ca^II^ and Zn^II^ ions are connected by two pairs of carboxylate ligands in *syn*±*syn*, and *syn*±*anti* bridging modes (Voegtli *et al.*, 2000). Another pair of carboxylates is coordinated with one Ca^II^ ion and two Zn^II^*via* (O,*O*') bridges. The distorted square pyramidal geometry around the Zn^II^ ion is completed by the coordination of N atoms of 4,4'-dimethyl-2,2'-bipyridine.

### 2. Experimental

4,4'-dimethyl-2,2'-bipyridine (0.5 g, 2.71 mmol) and zinc acetate (0.9 g, 4.1 mmol) of was added to hexa *N*-substituted (*p*-cyano benzamido) *para* xylyl based octa-aza cryptand (0.15 g) in 20 mL of ethanol-water (1:1, v/v) mixture at room temperature (Hossain *et al.*, 2010). The mixture was further diluted with 10 mL DMSO solvent with constant stirring. Only a few crystals were grown after a week, which was characterized by single-crystal diffraction method. Further analysis was not possible because of the small quantity of the product.

### 3. Refinement

H atoms on C were placed in idealized positions with C—H distances 0.95 - 0.98 Å and thereafter treated as riding. *U*_iso_ for H was assigned as 1.2 times *U*_eq_ of the attached atom (1.5 for methyl). The largest residual density peak was 1.50 Å from O2.

### Crystal data

**Table:**

[CaZn_2_(C_2_H_3_O_2_)_6_(C_12_H_12_N_2_)_2_]	*Z* = 1
*M**_r_* = 893.56	*F*(000) = 462
Triclinic, *P*1	*D*_x_ = 1.545 Mg m^−^^3^
Hall symbol: -P 1	Mo *K*α radiation, λ = 0.71073 Å
*a* = 8.3356 (10) Å	Cell parameters from 7990 reflections
*b* = 8.841 (1) Å	θ = 2.5–36.7°
*c* = 13.696 (2) Å	µ = 1.45 mm^−^^1^
α = 74.103 (7)°	*T* = 100 K
β = 83.263 (6)°	Parallelepiped, colorless
γ = 83.560 (7)°	0.34 × 0.32 × 0.27 mm
*V* = 960.6 (2) Å^3^	

### Data collection

**Table:**

Nonius KappaCCD diffractometer	8962 independent reflections
Radiation source: fine-focus sealed tube	7497 reflections with *I* > 2σ(*I*)
Graphite monochromator	*R*_int_ = 0.025
ω and φ scans	θ_max_ = 36.7°, θ_min_ = 3.0°
Absorption correction: multi-scan (*SCALEPACK*; Otwinowski & Minor, 1997)	*h* = −13→13
*T*_min_ = 0.639, *T*_max_ = 0.699	*k* = −14→14
16096 measured reflections	*l* = −22→22

### Refinement

**Table:**

Refinement on *F*^2^	Secondary atom site location: difference Fourier map
Least-squares matrix: full	Hydrogen site location: inferred from neighbouring sites
*R*[*F*^2^ > 2σ(*F*^2^)] = 0.034	H-atom parameters constrained
*wR*(*F*^2^) = 0.086	*w* = 1/[σ^2^(*F*_o_^2^) + (0.0386*P*)^2^ + 0.3887*P*] where *P* = (*F*_o_^2^ + 2*F*_c_^2^)/3
*S* = 1.05	(Δ/σ)_max_ = 0.001
8962 reflections	Δρ_max_ = 0.65 e Å^−^^3^
256 parameters	Δρ_min_ = −0.78 e Å^−^^3^
0 restraints	Extinction correction: *SHELXL97* (Sheldrick, 2008), Fc^*^=kFc[1+0.001xFc^2^λ^3^/sin(2θ)]^-1/4^
Primary atom site location: structure-invariant direct methods	Extinction coefficient: 0.0061 (13)

### Special details

**Table:**

Geometry. All e.s.d.'s (except the e.s.d. in the dihedral angle between two l.s. planes) are estimated using the full covariance matrix. The cell e.s.d.'s are taken into account individually in the estimation of e.s.d.'s in distances, angles and torsion angles; correlations between e.s.d.'s in cell parameters are only used when they are defined by crystal symmetry. An approximate (isotropic) treatment of cell e.s.d.'s is used for estimating e.s.d.'s involving l.s. planes.
Refinement. Refinement of *F*^2^ against ALL reflections. The weighted *R*-factor *wR* and goodness of fit *S* are based on *F*^2^, conventional *R*-factors *R* are based on *F*, with *F* set to zero for negative *F*^2^. The threshold expression of *F*^2^ > σ(*F*^2^) is used only for calculating *R*-factors(gt) *etc*. and is not relevant to the choice of reflections for refinement. *R*-factors based on *F*^2^ are statistically about twice as large as those based on *F*, and *R*- factors based on ALL data will be even larger.

### Fractional atomic coordinates and isotropic or equivalent isotropic displacement parameters (Å^2^)

**Table:**

	*x*	*y*	*z*	*U*_iso_*/*U*_eq_	
Zn1	0.645512 (17)	0.565205 (16)	0.245482 (10)	0.01344 (4)	
Ca1	0.5000	0.5000	0.5000	0.01472 (6)	
O1	0.42917 (11)	0.57148 (11)	0.33397 (7)	0.01780 (16)	
O2	0.36225 (13)	0.71366 (13)	0.18192 (8)	0.02391 (19)	
O3	0.83825 (11)	0.42210 (11)	0.30301 (7)	0.01864 (16)	
O4	0.69345 (13)	0.32456 (12)	0.45013 (8)	0.02265 (18)	
O5	0.71692 (12)	0.76819 (11)	0.26269 (7)	0.01935 (17)	
O6	0.68069 (13)	0.68704 (12)	0.43296 (7)	0.02151 (18)	
N1	0.73039 (13)	0.60239 (13)	0.09209 (8)	0.01657 (18)	
N2	0.56062 (13)	0.37004 (12)	0.20333 (8)	0.01583 (17)	
C1	0.81557 (16)	0.72407 (16)	0.04117 (10)	0.0197 (2)	
H1	0.8270	0.8035	0.0740	0.024*	
C2	0.88835 (16)	0.73989 (19)	−0.05764 (10)	0.0240 (3)	
C3	0.87138 (17)	0.62079 (19)	−0.10347 (10)	0.0252 (3)	
H3	0.9215	0.6255	−0.1701	0.030*	
C4	0.78117 (17)	0.49493 (18)	−0.05182 (10)	0.0222 (2)	
H4	0.7676	0.4143	−0.0832	0.027*	
C5	0.71088 (15)	0.48875 (15)	0.04678 (9)	0.0172 (2)	
C6	0.61311 (15)	0.35900 (15)	0.10892 (9)	0.0169 (2)	
C7	0.57756 (16)	0.23203 (17)	0.07454 (11)	0.0218 (2)	
H7	0.6131	0.2261	0.0071	0.026*	
C8	0.48959 (17)	0.11521 (16)	0.14069 (12)	0.0232 (3)	
H8	0.4667	0.0273	0.1189	0.028*	
C9	0.43442 (15)	0.12613 (15)	0.23931 (11)	0.0196 (2)	
C10	0.47342 (15)	0.25801 (14)	0.26603 (10)	0.0176 (2)	
H10	0.4362	0.2690	0.3322	0.021*	
C11	0.9812 (2)	0.8811 (2)	−0.11066 (13)	0.0346 (4)	
H11A	1.0352	0.8670	−0.1755	0.052*	
H11B	1.0625	0.8915	−0.0673	0.052*	
H11C	0.9061	0.9765	−0.1237	0.052*	
C12	0.34091 (18)	0.00266 (16)	0.31539 (13)	0.0256 (3)	
H12A	0.3132	0.0348	0.3787	0.038*	
H12B	0.4074	−0.0983	0.3295	0.038*	
H12C	0.2413	−0.0090	0.2876	0.038*	
C13	0.32312 (15)	0.64702 (15)	0.27164 (10)	0.0181 (2)	
C14	0.14820 (17)	0.6467 (2)	0.31439 (13)	0.0278 (3)	
H14A	0.0848	0.7359	0.2725	0.042*	
H14B	0.1400	0.6562	0.3845	0.042*	
H14C	0.1060	0.5478	0.3141	0.042*	
C15	0.82225 (15)	0.33229 (14)	0.39256 (10)	0.0172 (2)	
C16	0.97115 (18)	0.22946 (17)	0.43100 (12)	0.0258 (3)	
H16A	1.0392	0.2906	0.4569	0.039*	
H16B	1.0326	0.1928	0.3751	0.039*	
H16C	0.9382	0.1383	0.4860	0.039*	
C17	0.71988 (15)	0.78611 (14)	0.35138 (10)	0.0168 (2)	
C18	0.77759 (17)	0.93922 (15)	0.35734 (11)	0.0211 (2)	
H18A	0.7256	0.9663	0.4190	0.032*	
H18B	0.7491	1.0235	0.2972	0.032*	
H18C	0.8955	0.9271	0.3597	0.032*	

### Atomic displacement parameters (Å^2^)

**Table:**

	*U*^11^	*U*^22^	*U*^33^	*U*^12^	*U*^13^	*U*^23^
Zn1	0.01502 (6)	0.01454 (7)	0.01144 (6)	−0.00042 (4)	−0.00181 (4)	−0.00464 (4)
Ca1	0.01700 (14)	0.01496 (13)	0.01184 (13)	−0.00157 (10)	−0.00028 (10)	−0.00328 (10)
O1	0.0157 (4)	0.0207 (4)	0.0197 (4)	0.0005 (3)	−0.0035 (3)	−0.0098 (3)
O2	0.0267 (5)	0.0276 (5)	0.0201 (4)	−0.0007 (4)	−0.0052 (4)	−0.0103 (4)
O3	0.0182 (4)	0.0205 (4)	0.0170 (4)	0.0008 (3)	−0.0045 (3)	−0.0043 (3)
O4	0.0236 (4)	0.0183 (4)	0.0232 (5)	−0.0002 (3)	0.0002 (4)	−0.0024 (3)
O5	0.0234 (4)	0.0193 (4)	0.0172 (4)	−0.0026 (3)	−0.0031 (3)	−0.0072 (3)
O6	0.0267 (5)	0.0201 (4)	0.0180 (4)	−0.0057 (3)	−0.0054 (3)	−0.0026 (3)
N1	0.0152 (4)	0.0210 (5)	0.0138 (4)	0.0009 (3)	−0.0031 (3)	−0.0053 (3)
N2	0.0169 (4)	0.0158 (4)	0.0168 (4)	0.0015 (3)	−0.0045 (3)	−0.0076 (3)
C1	0.0180 (5)	0.0246 (6)	0.0152 (5)	−0.0017 (4)	−0.0014 (4)	−0.0032 (4)
C2	0.0177 (5)	0.0345 (7)	0.0161 (5)	0.0009 (5)	−0.0013 (4)	−0.0017 (5)
C3	0.0210 (6)	0.0391 (8)	0.0132 (5)	0.0048 (5)	−0.0015 (4)	−0.0058 (5)
C4	0.0209 (5)	0.0320 (7)	0.0149 (5)	0.0056 (5)	−0.0042 (4)	−0.0104 (5)
C5	0.0159 (5)	0.0235 (5)	0.0137 (5)	0.0044 (4)	−0.0050 (4)	−0.0080 (4)
C6	0.0154 (5)	0.0209 (5)	0.0166 (5)	0.0041 (4)	−0.0061 (4)	−0.0091 (4)
C7	0.0212 (6)	0.0259 (6)	0.0234 (6)	0.0042 (5)	−0.0075 (5)	−0.0152 (5)
C8	0.0211 (6)	0.0225 (6)	0.0322 (7)	0.0032 (4)	−0.0095 (5)	−0.0166 (5)
C9	0.0166 (5)	0.0166 (5)	0.0281 (6)	0.0024 (4)	−0.0072 (4)	−0.0097 (4)
C10	0.0171 (5)	0.0166 (5)	0.0211 (5)	0.0007 (4)	−0.0039 (4)	−0.0081 (4)
C11	0.0299 (7)	0.0446 (9)	0.0227 (7)	−0.0096 (7)	0.0040 (6)	0.0022 (6)
C12	0.0218 (6)	0.0177 (5)	0.0392 (8)	−0.0020 (4)	−0.0039 (5)	−0.0102 (5)
C13	0.0159 (5)	0.0188 (5)	0.0241 (6)	−0.0008 (4)	−0.0039 (4)	−0.0129 (4)
C14	0.0143 (5)	0.0355 (7)	0.0371 (8)	−0.0019 (5)	−0.0008 (5)	−0.0160 (6)
C15	0.0204 (5)	0.0133 (5)	0.0189 (5)	0.0013 (4)	−0.0063 (4)	−0.0053 (4)
C16	0.0263 (6)	0.0225 (6)	0.0279 (7)	0.0090 (5)	−0.0108 (5)	−0.0066 (5)
C17	0.0168 (5)	0.0155 (5)	0.0195 (5)	0.0000 (4)	−0.0049 (4)	−0.0063 (4)
C18	0.0259 (6)	0.0165 (5)	0.0234 (6)	−0.0039 (4)	−0.0062 (5)	−0.0067 (4)

### Geometric parameters (Å, º)

**Table:**

Zn1—O3	2.0243 (10)	C4—C5	1.3968 (18)
Zn1—O5	2.0334 (10)	C4—H4	0.9500
Zn1—O1	2.0556 (10)	C5—C6	1.4887 (19)
Zn1—N1	2.0851 (11)	C6—C7	1.4023 (18)
Zn1—N2	2.1741 (10)	C7—C8	1.388 (2)
Zn1—Ca1	3.4668 (5)	C7—H7	0.9500
Ca1—O4^i^	2.2876 (10)	C8—C9	1.400 (2)
Ca1—O4	2.2876 (10)	C8—H8	0.9500
Ca1—O6^i^	2.2910 (10)	C9—C10	1.3949 (17)
Ca1—O6	2.2910 (10)	C9—C12	1.504 (2)
Ca1—O1	2.3158 (10)	C10—H10	0.9500
Ca1—O1^i^	2.3158 (10)	C11—H11A	0.9800
Ca1—Zn1^i^	3.4668 (5)	C11—H11B	0.9800
O1—C13	1.2994 (16)	C11—H11C	0.9800
O2—C13	1.2313 (17)	C12—H12A	0.9800
O3—C15	1.2666 (15)	C12—H12B	0.9800
O4—C15	1.2520 (17)	C12—H12C	0.9800
O5—C17	1.2706 (15)	C13—C14	1.5061 (19)
O6—C17	1.2509 (16)	C14—H14A	0.9800
N1—C1	1.3386 (17)	C14—H14B	0.9800
N1—C5	1.3502 (16)	C14—H14C	0.9800
N2—C10	1.3401 (17)	C15—C16	1.5106 (18)
N2—C6	1.3415 (16)	C16—H16A	0.9800
C1—C2	1.3935 (19)	C16—H16B	0.9800
C1—H1	0.9500	C16—H16C	0.9800
C2—C3	1.393 (2)	C17—C18	1.5121 (17)
C2—C11	1.507 (2)	C18—H18A	0.9800
C3—C4	1.391 (2)	C18—H18B	0.9800
C3—H3	0.9500	C18—H18C	0.9800
			
O3—Zn1—O5	96.76 (4)	N2—C6—C5	114.92 (10)
O3—Zn1—O1	120.20 (4)	C7—C6—C5	124.11 (11)
O5—Zn1—O1	95.68 (4)	C8—C7—C6	118.89 (12)
O3—Zn1—N1	96.90 (4)	C8—C7—H7	120.6
O5—Zn1—N1	96.21 (4)	C6—C7—H7	120.6
O1—Zn1—N1	139.18 (4)	C7—C8—C9	120.39 (12)
O3—Zn1—N2	89.55 (4)	C7—C8—H8	119.8
O5—Zn1—N2	171.01 (4)	C9—C8—H8	119.8
O1—Zn1—N2	86.60 (4)	C10—C9—C8	116.57 (13)
N1—Zn1—N2	76.62 (4)	C10—C9—C12	120.25 (13)
O4^i^—Ca1—O4	180.0	C8—C9—C12	123.17 (12)
O4^i^—Ca1—O6^i^	86.56 (4)	N2—C10—C9	123.49 (12)
O4—Ca1—O6^i^	93.44 (4)	N2—C10—H10	118.3
O4^i^—Ca1—O6	93.44 (4)	C9—C10—H10	118.3
O4—Ca1—O6	86.56 (4)	C2—C11—H11A	109.5
O6^i^—Ca1—O6	180.0	C2—C11—H11B	109.5
O4^i^—Ca1—O1	93.28 (4)	H11A—C11—H11B	109.5
O4—Ca1—O1	86.72 (4)	C2—C11—H11C	109.5
O6^i^—Ca1—O1	97.73 (3)	H11A—C11—H11C	109.5
O6—Ca1—O1	82.27 (3)	H11B—C11—H11C	109.5
O4^i^—Ca1—O1^i^	86.72 (4)	C9—C12—H12A	109.5
O4—Ca1—O1^i^	93.28 (4)	C9—C12—H12B	109.5
O6^i^—Ca1—O1^i^	82.27 (3)	H12A—C12—H12B	109.5
O6—Ca1—O1^i^	97.73 (3)	C9—C12—H12C	109.5
O1—Ca1—O1^i^	179.999 (17)	H12A—C12—H12C	109.5
C13—O1—Zn1	105.63 (8)	H12B—C12—H12C	109.5
C13—O1—Ca1	146.51 (8)	O2—C13—O1	122.19 (12)
Zn1—O1—Ca1	104.79 (4)	O2—C13—C14	121.36 (13)
C15—O3—Zn1	119.31 (8)	O1—C13—C14	116.44 (12)
C15—O4—Ca1	136.47 (8)	C13—C14—H14A	109.5
C17—O5—Zn1	120.05 (8)	C13—C14—H14B	109.5
C17—O6—Ca1	140.65 (8)	H14A—C14—H14B	109.5
C1—N1—C5	119.60 (11)	C13—C14—H14C	109.5
C1—N1—Zn1	122.94 (9)	H14A—C14—H14C	109.5
C5—N1—Zn1	117.12 (9)	H14B—C14—H14C	109.5
C10—N2—C6	119.69 (11)	O4—C15—O3	124.57 (12)
C10—N2—Zn1	125.10 (8)	O4—C15—C16	118.87 (12)
C6—N2—Zn1	115.00 (9)	O3—C15—C16	116.56 (12)
N1—C1—C2	123.20 (13)	C15—C16—H16A	109.5
N1—C1—H1	118.4	C15—C16—H16B	109.5
C2—C1—H1	118.4	H16A—C16—H16B	109.5
C3—C2—C1	117.19 (13)	C15—C16—H16C	109.5
C3—C2—C11	122.54 (13)	H16A—C16—H16C	109.5
C1—C2—C11	120.27 (14)	H16B—C16—H16C	109.5
C4—C3—C2	120.09 (12)	O6—C17—O5	125.19 (11)
C4—C3—H3	120.0	O6—C17—C18	118.20 (11)
C2—C3—H3	120.0	O5—C17—C18	116.61 (11)
C3—C4—C5	119.08 (13)	C17—C18—H18A	109.5
C3—C4—H4	120.5	C17—C18—H18B	109.5
C5—C4—H4	120.5	H18A—C18—H18B	109.5
N1—C5—C4	120.82 (13)	C17—C18—H18C	109.5
N1—C5—C6	115.76 (10)	H18A—C18—H18C	109.5
C4—C5—C6	123.42 (12)	H18B—C18—H18C	109.5
N2—C6—C7	120.96 (12)		
			
O3—Zn1—O1—C13	−167.59 (7)	O1—Zn1—N2—C6	148.38 (9)
O5—Zn1—O1—C13	91.19 (8)	N1—Zn1—N2—C6	5.87 (8)
N1—Zn1—O1—C13	−15.15 (11)	C5—N1—C1—C2	−0.71 (19)
N2—Zn1—O1—C13	−80.09 (8)	Zn1—N1—C1—C2	172.43 (10)
Ca1—Zn1—O1—C13	165.81 (10)	N1—C1—C2—C3	−0.8 (2)
O3—Zn1—O1—Ca1	26.60 (5)	N1—C1—C2—C11	179.41 (13)
O5—Zn1—O1—Ca1	−74.63 (4)	C1—C2—C3—C4	1.7 (2)
N1—Zn1—O1—Ca1	179.04 (5)	C11—C2—C3—C4	−178.52 (14)
N2—Zn1—O1—Ca1	114.10 (4)	C2—C3—C4—C5	−1.1 (2)
O4^i^—Ca1—O1—C13	−12.60 (15)	C1—N1—C5—C4	1.28 (18)
O4—Ca1—O1—C13	167.40 (15)	Zn1—N1—C5—C4	−172.25 (9)
O6^i^—Ca1—O1—C13	74.37 (15)	C1—N1—C5—C6	−179.45 (11)
O6—Ca1—O1—C13	−105.63 (15)	Zn1—N1—C5—C6	7.02 (13)
O4^i^—Ca1—O1—Zn1	142.08 (4)	C3—C4—C5—N1	−0.37 (19)
O4—Ca1—O1—Zn1	−37.92 (4)	C3—C4—C5—C6	−179.58 (11)
O6^i^—Ca1—O1—Zn1	−130.95 (4)	C10—N2—C6—C7	0.24 (17)
O6—Ca1—O1—Zn1	49.05 (4)	Zn1—N2—C6—C7	175.22 (9)
O5—Zn1—O3—C15	103.30 (9)	C10—N2—C6—C5	−179.08 (10)
O1—Zn1—O3—C15	2.69 (10)	Zn1—N2—C6—C5	−4.10 (13)
N1—Zn1—O3—C15	−159.58 (9)	N1—C5—C6—N2	−1.71 (15)
N2—Zn1—O3—C15	−83.12 (9)	C4—C5—C6—N2	177.54 (11)
O6^i^—Ca1—O4—C15	165.69 (13)	N1—C5—C6—C7	178.99 (11)
O6—Ca1—O4—C15	−14.31 (13)	C4—C5—C6—C7	−1.76 (19)
O1—Ca1—O4—C15	68.13 (13)	N2—C6—C7—C8	−1.36 (18)
O1^i^—Ca1—O4—C15	−111.87 (13)	C5—C6—C7—C8	177.90 (12)
O3—Zn1—O5—C17	−68.30 (10)	C6—C7—C8—C9	1.38 (19)
O1—Zn1—O5—C17	53.08 (10)	C7—C8—C9—C10	−0.33 (19)
N1—Zn1—O5—C17	−166.04 (10)	C7—C8—C9—C12	−178.98 (12)
O4^i^—Ca1—O6—C17	−89.17 (14)	C6—N2—C10—C9	0.90 (18)
O4—Ca1—O6—C17	90.83 (14)	Zn1—N2—C10—C9	−173.54 (9)
O1—Ca1—O6—C17	3.67 (14)	C8—C9—C10—N2	−0.85 (18)
O1^i^—Ca1—O6—C17	−176.33 (14)	C12—C9—C10—N2	177.85 (12)
O3—Zn1—N1—C1	−92.32 (10)	Zn1—O1—C13—O2	−8.12 (14)
O5—Zn1—N1—C1	5.29 (10)	Ca1—O1—C13—O2	146.45 (12)
O1—Zn1—N1—C1	111.43 (10)	Zn1—O1—C13—C14	170.85 (9)
N2—Zn1—N1—C1	179.79 (11)	Ca1—O1—C13—C14	−34.6 (2)
O3—Zn1—N1—C5	80.97 (9)	Ca1—O4—C15—O3	−47.00 (19)
O5—Zn1—N1—C5	178.58 (9)	Ca1—O4—C15—C16	132.51 (12)
O1—Zn1—N1—C5	−75.27 (11)	Zn1—O3—C15—O4	−0.07 (17)
N2—Zn1—N1—C5	−6.91 (8)	Zn1—O3—C15—C16	−179.59 (9)
O3—Zn1—N2—C10	83.33 (10)	Ca1—O6—C17—O5	−35.1 (2)
O1—Zn1—N2—C10	−36.95 (10)	Ca1—O6—C17—C18	145.71 (11)
N1—Zn1—N2—C10	−179.46 (10)	Zn1—O5—C17—O6	−0.38 (18)
O3—Zn1—N2—C6	−91.33 (9)	Zn1—O5—C17—C18	178.85 (8)

Symmetry code: (i) −*x*+1, −*y*+1, −*z*+1.
